# Genomic Epidemiology of Global Carbapenemase-Producing *Escherichia coli,* 2015–2017

**DOI:** 10.3201/eid2805.212535

**Published:** 2022-05

**Authors:** Gisele Peirano, Liang Chen, Diego Nobrega, Thomas J. Finn, Barry N. Kreiswirth, Rebekah DeVinney, Johann D.D. Pitout

**Affiliations:** University of Calgary, Calgary, Alberta, Canada (G. Peirano, T.J. Finn, R. DeVinney, J.D.D. Pitout);; Alberta Precision Laboratories, Calgary (G. Peirano, J.D.D. Pitout);; Hackensack Meridian School of Medicine, Nutley, New Jersey, USA (L. Chen, B.N. Kreiswirth);; University of Guelph, Guelph, Ontario, Canada (D. Nobrega);; University of Pretoria, Pretoria, South Africa (J.D.D. Pitout)

**Keywords:** E. coli, surveillance, carbapenemases, molecular epidemiology, whole-genome sequencing, Escherichia coli, bacteria, antimicrobial resistance, AMR

## Abstract

We describe the global molecular epidemiology of 229 carbapenemase-producing *Escherichia coli* in 36 countries during 2015–2017. Common carbapenemases were oxacillinase (OXA) 181 (23%), New Delhi metallo-β-lactamase (NDM) 5 (20%), OXA-48 (17%), *Klebsiella pneumoniae* carbapenemase 2 (15%), and NDM-1 (10%). We identified 5 dominant sequence types (STs); 4 were global (ST410, ST131, ST167, and ST405), and 1 (ST1284) was limited to Turkey. OXA-181 was frequent in Jordan (because of the ST410-B4/*H*24RxC subclade) and Turkey (because of ST1284). We found nearly identical IncX3-*bla*_OXA-181_ plasmids among 11 STs from 12 countries. NDM-5 was frequent in Egypt, Thailand (linked with ST410-B4/*H*24RxC and ST167-B subclades), and Vietnam (because of ST448). OXA-48 was common in Turkey (linked with ST11260). Global *K. pneumoniae* carbapenemases were linked with ST131 C1/*H*30 subclade and NDM-1 with various STs. The global carbapenemase *E. coli* population is dominated by diverse STs with different characteristics and varied geographic distributions, requiring ongoing genomic surveillance.

## Abstract 2

The *E. coli* population is dominated by diverse sequence types with varied geographic distributions, warranting ongoing genomic surveillance.

Carbapenems are effective options available for treating serious infections caused by multidrug-resistant (MDR) Enterobacterales bacteria (*1*). The emergence of carbapenem resistance is a major public health concern, and the World Health Organization has identified carbapenem-resistant Enterobacterales as critical-priority bacteria (*2*).

Carbapenemases are important causes of carbapenem resistance (*3*). Carbapenemase genes can be transferred between Enterobacterales species. The most common carbapenemases among Enterobacterales are *Klebsiella pneumoniae* carbapenemases (KPCs), imipenemases (IMPs), Verona integron–encoded metallo-β-lactamases (VIMs), New Delhi metallo-β-lactamases (NDMs), and oxacillinase (OXA) 48–like enzymes. *Escherichia coli* is the second most common carbapenemase-producing Enterobacterales species (*4*,*5*).

Because *E. coli* is mainly responsible for human community-associated infections (*6*), it evades conventional hospital-based infection-prevention measures (*7*). *E. coli* is an important One Health (i.e., human, animal, environmental health) reservoir for antimicrobial resistance (AMR) genes (*8*). Tracking global mobile genetic elements and *E. coli* clones associated with carbapenemase genes is a public health priority (*9*) and aids in designing management and prevention strategies.

Comprehensive epidemiology data about carbapenemase-producing *E. coli* is limited to institutional, regional, or countrywide surveys (*10*). We used short-read whole-genome sequencing (WGS) to describe the molecular characteristics and international distribution of carbapenemase-producing *E. coli*. We describe the geographic distribution of different carbapenemase genes (including their associations with dominant sequence types [STs], clades and underlying mobile genetic elements), other β-lactamases, AMR genes, and virulence factors.

## Materials and Methods

### Bacterial Isolates

We obtained ethics approval for this study through the University of Calgary Conjoint Health Research Ethics Board (approval no. REB17-1010). We included 229 clinical, nonrepeat *E. coli* isolates collected from 2 global surveillance programs (SMART and INFORM) during 2015–2017 (Appendix). Isolates had undergone identification and susceptibility testing using Clinical Laboratory and Standards Institute guidelines (*4*,*5**,**11*). Carbapenem nonsusceptible isolates underwent molecular screening for *bla*_KPC_, *bla*_VIM_, *bla*_NDM_, *bla*_OXA-48-like_, *bla*_IMP_, and *bla*_GES_, as described previously (*4*,*5*). Overall, we collected 87,182 Enterobacterales for the period 2015–2017 from 62 countries: 27,444 were identified as *E. coli* and 275 (1%) tested nonsusceptible to >1 of the carbapenems. Most (229 [83%]) were positive for either *bla*_KPC_, *bla*_OXA-48-like_, *bla*_NDM_, *bla*_VIM_, or *bla*_IMP_ and were included in this study. The remaining 46 were negative for *bla*_KPC_, *bla*_VIM_, *bla*_NDM_, *bla*_OXA-48-like_, *bla*_IMP_, and *bla*_GES_.

We defined major STs as representing >10% and minor STs as representing 5%–10% of the total *E. coli* carbapenemase population (*12*). Dominant STs were both major and minor STs.

### Genomic Analysis

We subjected the carbapenemase-producing *E. coli* (n = 229) to short-read WGS by using NovoSeq (Illumina, https://www.illumina.com) with 151 × 2 paired-end reads (*13*,*14*). We obtained draft genomes by using SPAdes 3.15 (*15*). We used BLAST (https://blast.ncbi.nlm.nih.gov/Blast.cgi) to determine AMR genes, plasmid replicons, and virulence genes against the following databases or typing schemes: National Center for Biotechnology Information Bacterial Antimicrobial Resistance Reference Gene Database (https://www.ncbi.nlm.nih.gov/bioproject/PRJNA313047), ResFinder (*16*), PlasmidFinder (*17*), multilocus sequence typing (*18*), and virulence finder (*19*). We conducted multilocus sequence typing by using *mlst* 2.19 (https://github.com/tseemann/mlst). We identified ST410 and ST131 clades and subclades as described previously (*20*,*21*).

For phylogenetic analyses, we mapped trimmed raw reads from each genome to a reference genome sequence (EC958 [GenBank accession no. HG941718] for ST131, JS316 [GenBank accession no. CP058618] for ST410, WCHEC005237 [GenBank accession no. CP026580] for ST167, and AR_0015 [GenBank accession no. CP024862] for ST405) by using snippy (https://github.com/tseemann/snippy). We filtered single-nucleotide polymorphisms (SNPs) among prophages, repeated sequences, or insertion sequences as previously described (*22*), and we generated a maximum-likelihood phylogenetic tree inferred from the resulting SNP alignment by using RAxML 8.2.12 by using a general time-reversible model of nucleotide substitution and 4 discrete γ categories of rate heterogeneity (*23*). We identified phylogenetic clades by using hierarchical Bayesian analysis of the population structure in R by using RhierBAPS with 10 initial clusters at 2 clustering levels (*24*). We defined clades by using the first level of clustering and subclades at the second level of clustering (*25*). We annotated the phylogenetic trees in iTOL (*26*). We deposited all sequencing data in the National Center for Biotechnology Information database (BioProject PRJNA780590).

### Statistical Analyses

We conducted all analyses in R 3.6.1 (*27*). Initially, we attempted to fit generalized linear mixed models with country-level random effects to summarize comparisons between dominant STs with respect to antimicrobial and virulence genes. Most models failed to converge, possibly because of the low number of isolates for some STs and the large number of countries involved. Thereafter, we attempted to use exact logistic regression models for clustered data, as previously described (*28*). Similarly, most models failed to converge, particularly for comparisons involving ST1284 where all isolates were obtained from a single country. We then used Fisher exact tests to perform pairwise comparisons of antimicrobial and virulence genes among dominant STs. We used Mann-Whitney tests for comparison of virulence scores between dominant STs. We adjusted p values for multiple comparisons within each outcome by using the false discovery rate (*29*). We defined statistical significance as p>0.05.

## Results

### Global Distribution of Carbapenemases

Overall, 218 isolates were positive for a single carbapenemase and 11 isolates were positive for 2 carbapenemases (Table). The OXA-48–like (n = 106) were the most common carbapenemases, followed by NDMs (n = 77), KPCs (n = 50), VIMs (n = 5), and IMPs (n = 2). The OXA-48–like carbapenemases consisted of OXA-48 (n = 41), OXA-181 (n = 55), OXA-244 (n = 3), and OXA-232 (n = 7). *E. coli* with OXA-48, OXA-181, and OXA-232 had a global distribution. OXA-244 was limited to Egypt (Table). The NDMs consisted of NDM-1 (n = 22), NDM-4 (n = 1), NDM-5 (n = 48), NDM-6 (n = 1), and NDM-7 (n = 5). *E. coli* with NDM-1 and NDM-5 had a global distribution. NDM-4 was limited to Vietnam and NDM-6 to Guatemala; NDM-7 was found in the Philippines and Vietnam (Table). The KPCs consisted of KPC-2 (n = 35), KPC-3 (n = 14), and KPC-18 (n = 1). *E. coli* with KPC-2 and KPC-3 had a global distribution, and KPC-18 was obtained from the United States (Table). *E. coli* with VIMs (VIM-1 and VIM-23) were found in Greece, Spain, Mexico, and Egypt; *E. coli* with IMP-59 were obtained from Australia (Table).

**Table:** Global molecular epidemiology of 229 carbapenemase-producing *Escherichia coli* isolates, 36 countries, 2015–2017*

Carbapenemases (no. isolates)	Geographic location (no. isolates)	Sequence types (no. isolates)
KPCs (50)		
KPC-2 (35)	Argentina (4), Brazil (5), Colombia (8), Greece (1), Guatemala (4), Israel (2), Puerto Rico (2), United States (4), Venezuela (1), Vietnam (4)	ST10 (3), ST46 (2), ST69 (2), ST95 (3), ST131 (7), ST349 (1), ST405 (3), ST410 (3), ST538 (1), ST540 (1), ST607 (1), ST617 (1), ST648 (1), ST1193 (1), ST1196 (1), ST2172 (1), ST2279 (1), ST3580 (1)
KPC-3 (14)	Colombia (1), Israel (1), Italy (8), United States (4)	ST12 (1), ST73 (1), ST131 (7), ST141 (1), ST191 (1), ST617 (1), ST973 (1), ST1148 (1)
KPC-18 (1)	United States (1)	ST131 (1)
NDMs (66)		
NDM-1 (19)	Egypt (3), Guatemala (2), Kuwait (1), Morocco (4), Philippines (1), Romania (1), Russia (3), Serbia (1), Thailand (2), Vietnam (1)	ST38 (1), ST44 (1), ST69 (1), ST95 (1), ST131 (4), ST167 (3), ST345 (1), ST361 (1), ST617 (2), ST1193 (1), ST1434 (1), ST1470 (1), ST4553 (1),
NDM-4 (1)	Vietnam (1)	ST405 (1)
NDM-5 (40)	Canada (1), Egypt (16), Italy (2), Jordan (4), Lebanon (1), Thailand (8), United Kingdom (2), Vietnam (6)	ST131 (1), ST156 (1), ST167 (11), ST361 (4), ST405 (3), ST410 (12), ST448 (2), ST648 (4), ST2003 (2)
NDM-6 (1)	Guatemala (1)	ST38 (1)
NDM-7 (5)	Philippines (4), Vietnam (1)	ST156 (2), ST410 (1), ST448 (1), ST5229 (1)
OXA-48–like (96)		
OXA-48 (40)	Austria (1), Belgium (2), Egypt (3), Georgia (3), Israel (1), Lebanon (2), Mexico (1), Morocco (2), Saudi Arabia (1), Spain (2), Thailand (1), Tunisia (1), Turkey (15), United Kingdom (1), Vietnam (4)	ST10 (2), ST12 (1), ST34 (1), ST38 (8), ST58 (1), ST131 (2), ST224 (1), ST349 (1), ST354 (6), ST361 (1), ST405 (4), ST410 (2), ST624 (1), ST648 (1), ST1431 (1), ST11260 (6)
OXA-181 (48)	Egypt (6), Germany (1), Jordan (15), Kuwait (1), Lebanon (1), Malaysia (1), South Africa (2), Taiwan (1), Thailand (2), Turkey (18)	ST46 (1), ST131 (1), ST167 (2), ST205 (1), ST354 (1), ST410 (21), ST648 (1), ST1284 (18), ST1487 (1), ST6802 (1)
OXA-232 (5)	Malaysia (1), Mexico (3), Thailand (1)	ST127 (1), ST131 (1), ST361 (3)
OXA-244 (3)	Egypt (3)	ST58 (1), ST648 (1), ST1722 (1)
VIMs (4)		
VIM-1 (2)	Greece (1), Spain (1)	ST88 (1), ST404 (1)
VIM-23 (2)	Mexico (2)	ST410 (2)
IMPs (2)		
IMP-59 (2)	Australia (2)	ST357 (2)
Two carbapenemases (11)		
NDM-1 + VIM-1 (1)	Egypt (1)	ST131 (1)
NDM-1 + OXA-181 (2)	Egypt (2)	ST46 (2)
NDM-5 + OXA-48 (1)	Egypt (1)	ST167 (1)
NDM-5 + OXA-181 (5)	Egypt (3), South Korea (1), Vietnam (1)	ST410 (4), ST448 (1)
NDM-5 + OXA-232 (2)	United Kingdom (2)	ST2083 (2)

*KPC, *Klebsiella pneumoniae* carbapenemase; NDM, New Delhi metallo-β-lactamase; OXA, oxacillinase; ST, sequence type.

### Global Distribution of Dominant *E. coli* Sequence Types and Clades

We identified 2 major STs (ST410 [20%] and ST131 [12%]) and 3 minor STs (ST1284 [8%], ST167 [7%], and ST405 [5%]) among this collection. The next most common STs did not fulfill the definition of a dominant ST: ST38 (n = 10 [4%]), ST354 (n = 7 [3%]), ST361 (n = 9 [4%]), ST648 (n = 8 [4%]), and ST11260 (n = 6 [3%]).

ST410 was the most common ST (n = 45/229 [20%]) and was positive for KPC-2 (7%), NDM-5 (27%), NDM-7 (2%), OXA-48 (4%), OXA-181 (47%), and VIM-23 [4%]) (Appendix Table 1). ST410 belonged to 2 subclades: B3/*H24*Rx (n = 10) and B4/*H24*RxC (n = 35) (*21*).

ST131 was the second most common ST (n = 26/229 [12%]) and was positive for KPC-2 (n = 8), KPC-3 (n = 7), KPC-18 (n = 1), NDM-1 (n = 5), NDM-5 (n = 1), OXA-48 (n = 2), OXA-181 (n = 1), and OXA-232 (n = 1). One NDM-1 isolate was also positive for VIM-1. ST131 belonged to clade A/*H*41 (n = 2) and subclades C1_nonM27 (n = 10), C1_M27 (n = 4), and C2 (n = 10). We also note the global distribution of different minor STs (ST1284, ST167, ST405) and their clades (Appendix).

### AMR Determinants and Plasmid Replicon Types

We determined quinolone resistance–determining regions mutations, β-lactamases (noncarbapenemases), aminoglycoside modifying enzymes, and plasmid replicon types among the different *E. coli* STs (Appendix Table 1). TEM-1, CTX-M-15, *aac(6')-Ib-cr*, and *sul1* were common among isolates.

### Virulence Associated Factors

We assessed the presence of 37 putative virulence factors among the different dominant STs (Appendix Table 2). The following factors were present among most of isolates: *fimH* (100%), *fyuA* (55%), *traT* (64%), and *iss* (52%). Some virulence factors were associated with certain STs: *papA* (81%), *iha* (77%), *sat* (81%), *fyuA* (100%) *usp* (100%), *ompT* (100%), and *malX* (100%) with ST131, and *astA* (100%) and *iutA* (100%) with ST1284. ST131 had the highest overall number of virulence genes (n = 11), and ST410 had the lowest number of virulence genes (n = 2) (Appendix Table 2).

### Carbapenemase Gene Flanking Regions and Plasmid Analysis

Because of the limitations of short-read sequencing (*30*), analyses of the immediate carbapenemase gene flanking regions and plasmids harboring carbapenemase genes were insufficient, especially for *bla*_OXA-48_ and *bla*_VIMs_. We obtained results for 20/22 of *bla*_NDM-1_, 2/2 of *bla*_NDM-4_, 46/48 of *bla*_NDM-5_, 1/1 of *bla*_NDM-6_, 4/5 of *bla*_NDM-7_, 34/35 of *bla*_KPC-2_, 14/14 of *bla*_KPC-3_, 1/1 of *bla*_KPC-18_, 1/41 *bla*_OXA-48_, 55/55 of *bla*_OXA-181_, 3/3 of *bla*_OXA-244_, and 7/7 of *bla*_OXA-232_.

Among *bla*_KPC-2_, 15 were situated in Tn*4401* elements (Tn*4401a* [n = 4] in ST131 and ST46, Tn*4401b* [n = 9] in 7 STs, and Tn*4401e* [n = 2] in ST131 and ST1193). Nineteen were associated with non-Tn*4401* mobile elements (NTM_KPC_) (*31*), including 4 ST131 and 3 ST405 strains. The *bla*_KPC-3_ genes were associated with Tn*4401a* (n = 9), Tn*4401b* (n = 3), and Tn*4401d* (n = 2). The *bla*_KPC-18_ was located on a novel Tn*4401* variant (186 bp deletion). The *bla*_NDM_s were located on truncated Tn*125* elements, and the *bla*_NDM_ upstream regions showed substantial diversities with various IS element insertions (e.g., IS*630*, IS*Aba125*, IS*1*, and IS*903* with *bla*_NDM-1_; IS*Ecp1* and IS*1* with *bla*_NDM-5_; and IS*5* with *bla*_NDM-7_). 

All *bla*_OXA-232_ genes were located on the same 6.1 kb colKp3 plasmids (pOXA-232) (*32*). Sequence similarities (95%–100%) of *bla*_KPC-3_ isolates with previously sequenced plasmids in GenBank showed that most (n = 9) were harbored within IncFIBQil plasmids (*33*); 2 KPC-3 genes were within pKPC-CAV1193 (*34*), 1 *bl*a_KPC-3_ was within the IncFIA plasmid pBK30683 (*35*), and 1 *bl*a_KPC-3_ was in the IncI2 plasmid pBK15692 (*36*). The *bla*_OXA-181_ (n = 55) were situated within Tn*2013* harbored on the identical IncX3 plasmids with 99%–100% similarities to plasmid p72_X3_OXA181 (*37*). p72_X3_ OXA181 contained the IncX3 and truncated ColKp3 replicons (*13*).

## Discussion

A World Health Organization report showed the lack of adequate surveillance programs in many parts of the world, especially from lower- and middle-income countries (LMICs) (*38*). That report identified bacteria, including carbapenem-resistant *E. coli,* where global surveillance data are urgently required. LMICs bear a considerable share of the disease burden attributable to MDR *E. coli* but lack adequate genomic surveillance systems (*39*). Our study aimed to describe the global molecular epidemiology of 229 carbapenemase-producing *E. coli* obtained from 36 countries (including 20 LMICs) during 2015–2017. Isolates with multiple AMR genes dominated the population. The most common carbapenemase group was the OXA-48-like carbapenemases (44%), followed by NDMs (32%), KPCs (21%), VIMs (2%), and IMPs (1%). OXA-48-like carbapenemases were numerous in Egypt, Jordan, and Turkey; NDMs were numerous in Egypt, Thailand, and Vietnam, and KPCs were numerous in Colombia, Italy, and the United States.

We identified 5 dominant STs and their respective clades and subclades; 4 were global: ST410 subclades B3/*H24*Rx and B4/*H24*RxC; ST131 clade A/*H*41, subclades C1_nonM27/*H*30, C1_M27/*H*30, and C2/*H*30; ST167 subclades B1, B2, and B3; and ST405 clades A and B (Appendix Figure). ST1284 (1 clade) was limited to Turkey, and the ST167-A clade was limited to Guatemala. Dominant STs and their respective clades and subclades were associated with different underlying mobile genetic elements: ST410 was linked with NDM-5 and OXA-181; ST131 was linked with KPCs, ST1284 was linked with OXA-181, ST167 was linked with NDM-5, and ST405 was linked with various carbapenemases.

A recent survey of global carbapenemase-producing *E. coli* for the period 2002–2017 included 343 carbapenem-resistant isolates obtained mainly from the United States (*40*). KPC (16%), NDM (16%), and OXA-48–like (13%) carbapenemases were common. The study screened for different *E. coli* phylogroups and certain STs (ST131, ST648, and ST405). Phylogroup B2 isolates were common, and phylogroup A was dominant in Asia. Global ST131 with *bla*_KPC_s was the most common ST, followed by ST648 with *bla*_OXA-48-like_ and ST405 with *bla*_NDM_s.

The most frequent individual carbapenemases in our survey were OXA-181 (23%), NDM-5 (20%), OXA-48 (17%), KPC-2 (15%), and NDM-1 (10%). This result was different from carbapenemase-producing *K. pneumoniae* and *Enterobacter cloacae* complex with carbapenemases obtained from the same surveillance programs (*14*,*41*). The *K. pneumoniae* population was dominated by ST258 with KPC-2 from Greece and KPC-3 from the United States (*41*). The *E. cloacae* complex isolates (various STs) were dominated by VIM-1 from Greece and Italy (*14*). *K. pneumoniae* (*42*) and *E. cloacae* complex (*43*) are mainly hospital pathogens, whereas *E. coli* was mainly a community pathogen (*6*), which could partly be responsible for the different carbapenemase types among these species.

Molecular-based surveillance studies have shown that OXA-48–like enzymes are common among global carbapenemase-producing Enterobacterales (*4*,*5*). OXA-48 is currently the most common OXA-48–like derivative and OXA-181 the second most common derivative (*44*). OXA-48 is endemic in North Africa, Middle East, and Turkey (*44*). *E. coli* with *bla*_OXA-48_ is linked to various STs (*44*). In our study, OXA-48 was identified among 18 STs from 15 countries. *E. coli* with *bla*_OXA-48_ was common in Turkey, where it was linked with ST11260.

OXA-181 is linked with certain *E. coli* STs, especially ST410 (*44*). *E. coli* ST410 belongs to phylogroup A and is divided into 2 clades (A/*H*53 and B/*H*24). Clade B is divided into subclades B1/*H*24, B2/*H*24R, B3/*H*24Rx, and B4/*H*24RxC (*21*). The B2/*H*24R subclade is associated with fluroquinolone resistance, B3/*H*24Rx with *bla*_CTX-M-15_, and B4/*H*24RxC with *bla*_OXA-181_ (*21*). In our survey, OXA-181 was identified among 11 different STs obtained from 12 countries. All the OXA-181 genes were situated within Tn*2013* harbored on near identical IncX3 plasmids (≈100% similarly to p72_X3_OXA181). *K. pneumoniae* ST307 with p72_X3_OXA181 was previously responsible for large outbreaks in South Africa (*13*,*37*). *E. coli* with *bla*_OXA-181_ was frequent in Jordan, Egypt (linked with ST410- B4/*H24*RxC subclade), and Turkey (linked with ST1284). The ST410-B4/*H24*RxC subclade with *bla*_OXA-181_ was also found in Thailand and South Korea. The ST410-B3/*H24*Rx subclade with *bla*_OXA-181_ was present in South Africa and Kuwait.

Molecular-based surveillance studies have shown that NDMs are often the most common carbapenemase in certain regions (e.g., the Indian subcontinent) (*4*,*5*). NDM-1 is the most frequent NDM enzyme and associated with various STs within diverse plasmid platforms (*45*). In our survey, NDM-1 was identified among 14 different STs obtained from 10 countries. *E. coli* with *bla*_NDM-1_ was not linked with a specific ST and was evenly distributed among the different countries.

*E. coli* with NDM-5 is numerous among *E. coli* with NDMs from India, China, and sub-Saharan Africa (*45*). NDM-5, in our survey, was found among 9 different STs from 9 countries. It was common in Egypt (linked with ST410 [B4/*H24*RxC] and ST167 [B1 and B3]), Thailand (linked with ST410 [B4/*H24*RxC] and ST167 [B2]), and Vietnam (linked with ST448). ST167 belongs to phylogroup A and is an emerging carbapenemase clone associated with *bla*_NDM-5_ (*46*). We divided ST167 into 2 clades (A and B) and 3 subclades (B1, B2, and B3). Subclade B3 was the most dominant clade and associated with *bla*_NDM-5_ obtained from Egypt and Italy. Other subclades were less common and linked with *bla*_NDM-5_, *bla*_NDM-1_, and *bla*_OXA-181_ obtained in Guatemala (clade A), Egypt (subclades B1 and B2), and Thailand (subclade B2).

*E. coli* with *bla*_KPC_ is associated with ST131 (*47*) on diverse plasmid platforms (*48*). *E. coli* ST131 is global MDR high-risk clone associated with fluoroquinolone resistance and *bla*_CTX-M_s (*49*). ST131 belongs to clades A/*H*41, B/*H*22, and C/*H*30 (*50*). C/*H*30 is divided into subclades C0, C1_nonM27, C1_M27, and C2. In our survey, KPC genes were found among 26 different STs from 11 countries. *E. coli* with *bla*_KPC_ was common in Colombia linked with various STs. ST131 was responsible for 32% of KPC isolates and obtained from Italy, Israel, Guatemala, Puerto Rico, and the United States (including Puerto Rico). ST131 with *bla*_KPC_ was dominated by the C1_nonM27 subclade. This dominance is different from that observed by Johnson et al. study (*40*), where the C2 subclade was common. The ST131-C1_M27 subclade in our survey was positive for *bla*_NDM-1_ and *bla*_OXA-232_.

Among this study’s strengths is that it included a large global collection of recent isolates representing multiple LMICs. We characterized all isolates using short-read WGS and provided novel information regarding the geographic distribution and MDR determinants of dominant STs and their respective clades and subclades (e.g., global ST410 was linked with *bla*_OXA-181_, ST131 with *bla*_KPCs_, ST167 with *bla*_NDM-5_, and ST405 with various carbapenemases).

We showed that the underlying molecular epidemiology within the same carbapenemase groups were very different (e.g., NDM-1 was linked with various STs, including ST131-C2/*H*30, whereas NDM-5 was linked with ST167-B and ST410B4/*H*24Rx). The geographic distribution of isolates with NDM-1 and NDM-5 was different (e.g., NDM-1 showed global distribution whereas those with NDM-5 were numerous in Egypt, Thailand, and Vietnam). Similar differences were described for isolates with OXA-48 (various STs) and OXA-181 (linked with ST410B4/*H*24Rx). Future genomic surveys should use methodologies that characterize individual carbapenemases.

We also showed that global *bla*_OXA-181_ was harbored on near identical IncX3 plasmids (irrespective of the ST or geographic location). This finding suggests that highly similar IncX3 plasmids were mainly responsible for the global distribution of OXA-181 genes, the most common carbapenemase in this collection. The control of such IncX3 plasmids should be a public health priority.

Limitations of this study include the fact that flanking regions and plasmids harboring carbapenemases were not fully reconstructed because of the limitations of short-read sequencing (*30*). The characterization of plasmids is vital to fully comprehend the molecular epidemiology of global carbapenemase-producing *E. coli*, and a follow-up study using long-read sequencing is under way. Several countries included only few isolates (Table) and therefore may not be fully representative of what carbapenemase-producing *E. coli* dominates in that region.

In summary, the global carbapenemase-producing *E. coli* population is dominated by diverse STs with different characteristics and varied geographic distributions. This characterization was especially apparent within certain carbapenemases groups (i.e., NDM-1 vs. NDM-5 or OXA-48 vs. OXA-181). Ongoing genomic surveillance to characterize individual carbapenemases will assist in designing management and prevention strategies to help curtail the spread of AMR bacteria.

Appendix Additional information about genomic epidemiology of global carbapenemase-producing *Escherichia coli,* 2015–2017.
