# Supplementary material for: Genomic Epidemiology of Global Carbapenemase-Producing Escherichia coli, 2015–2017
Source: Emerg Infect Dis. 2022 May;28(5):924–31. doi: 10.3201/eid2805.212535 (PMC9045447; doi:10.3201/eid2805.212535)
Supplement: Appendix — Additional information about genomic epidemiology of global carbapenemase-producing Escherichia coli, 2015–2017. [file 21-2535-Techapp-s1.pdf]

Article DOI: https://doi.org/10.3201/eid2805.212535

Genomic Epidemiology of Global Carbapenemase-Producing *Escherichia coli,* 2015–2017

Appendix

Details of the SMART and INFORM surveillance programs

The Merck Study for Monitoring Antimicrobial Resistance Trends (SMART) program (2015–17) included isolates from intra-abdominal and urinary tract infections from the following 55 countries: Egypt, Morocco, Kenya, South Africa, Tunisia (Africa); China, Hong Kong, India, Japan, Malaysia, Singapore, South Korea, Taiwan, Thailand, Vietnam (Asia); Croatia, Czech Republic, Estonia, France, Georgia, Greece, Germany, Hungary, Italy, Latvia, Lithuania, Portugal, Romania, Serbia, Slovenia, Spain, Switzerland, Turkey, United Kingdom (Europe); Argentina, Brazil, Chile, Colombia, Dominican Republic, Ecuador, Guatemala, Mexico, Puerto Rico, Panama, Uruguay, Venezuela (Latin America); Jordan, Lebanon, Israel, Saudi Arabia, UAE (Middle East); Canada, United States (North America); and Australia, New Zeeland, Philippines (South Pacific).

The INFORM surveillance program of antimicrobial resistance (2015–17) included isolates from intra-abdominal, urinary tract, blood, skin and soft tissue and lower respiratory tract infections from the following 42 countries: Egypt, Kenya, Nigeria, South Africa (Africa); China, South Korea, Taiwan, Thailand (Asia); Austria, Belgium, Bulgaria, Greece, Czech Republic, Denmark, France, Germany, Hungary, Italy, Macedonia, Portugal, Poland, Russia, Romania, Slovakia, Spain, Turkey, United Kingdom (Europe); Argentina, Brazil, Chile, Colombia, Mexico, Uruguay, Venezuela (Latin America); Lebanon, Israel, Syria, Kuwait (Middle East); United States (North America); and Australia, Philippines, Japan (South Pacific).

Both programs collected 100 consecutive clinically relevant non-repeat Gram-negative bacteria per annum from each institution.

Global distribution of dominant *E. coli* Sequence types (STs) and clades

ST410

The ST410-B3/*H24*Rx subclade showed a global distribution (Georgia [n = 1] with OXA-48, Kuwait [n = 1] with OXA-181, Mexico [n = 2] with VIM-23, Morocco [n = 1] with OXA-48, South Africa [n = 2] with OXA-181, USA [n = 2] with KPC-2 and Vietnam [n = 1] with KPC-2). The ST410-B4/*H24*RxC subclade was found in Jordan (n = 15 with OXA-181), Egypt (n = 11 with NDM-5 and OXA-181), Philippines (n = 1 with NDM-7), South Korea (n = 1 with NDM-5 and OXA-181), Thailand (n = 6 with NDM-5 and OXA-181 and Vietnam (n = 1 with NDM-5).

ST131

The ST131-A/*H*41 clade (n = 2) was positive for KPC-3 (from USA) and OXA-48 (from Lebanon). The ST131-C1_M27/*H*30 subclade (n = 4) was positive for NDM-1 (from Russia (n = 2), Philippines [n = 1]) and OXA-232 from Thailand (n = 1). The ST131-C1_nonM27/*H*30 subclade (n = 10) was positive for KPC-2 from Guatemala (n = 4), Israel (n = 2) and USA (n = 1); KPC-3 from Italy (n = 2) and KPC-18 from USA (n = 1). The ST131-C2/*H*30 subclade (n = 10) was positive for KPC-2 (n = 1 from Puerto Rico), KPC-3 (n = 4 from Italy), NDM-1 (n = 2 from Egypt), NDM-5 (n = 1 from Canada), OXA-48 (n = 1 from Egypt), and OXA-181 (n = 1 from Egypt). One ST131-C2 with NDM-1 was also positive for VIM-1.

ST167

The ST167-A clade (n = 2) was found in Guatemala with NDM-1, ST167-B1 subclade (n = 3) was found in Egypt with NDM-5; ST167-B2 subclade (n = 5) was found in Egypt (n = 2 with OXA-181), and Thailand (n = 3 with NDM-5); ST167-B3 subclade (n = 7) was present in Egypt (with NDM-1 [n = 1], NDM-5 [4]) and Italy (n = 2 with NDM-5).

ST405

The ST405-A clade was found in Egypt with NDM-5 and OXA-48. The ST405-B clade was present in Colombia (n = 1 with KPC-2), Spain (n = 1 with OXA-48), Vietnam (n = 4 with NDM-5 and KPC-2), Turkey (n = 2 with OXA-48) and UK (n = 1 with NDM-5.

The non-dominant STs (n = 112/229 [49%]) were positive for KPC-2, KPC-3, NDM-1, NDM-5, NDM-6, NDM-7, OXA-48, OXA-181, OXA-232, OXA-244, VIM-1 and IMP-59

(Appendix Table 1). Non-dominant STs showed a global distribution. They were found in Europe (Austria, Belgium, Georgia, Germany, Greece, Italy, Romania, Russia, Serbia, Spain, UK, Turkey), Africa (Egypt, Morocco, Tunisia), Middle East (Lebanon, Israel, Jordan, Kuwait, Saudi Arabia), Asia (Malaysia, Thailand, Philippines, Taiwan, Vietnam), Latin America (Argentina, Brazil, Colombia, Guatemala, Mexico, Puerto Rico, Venezuela), North America (USA) and Australia. Non-dominant STs were common in Turkey where they were linked with OXA-48; in Egypt where they were linked with NDM-1, NDM-5, OXA-181, OXA-244, and OXA-48; and Vietnam where they were linked with KPC-2, NDM-1, NDM-5, NDM-7, OXA-48 and OXA-181.

Appendix Table 1. Characteristics of dominant Sequence Types among carbapenemase-producing *Escherichia coli*

Characteristic	ST410 n = 45 (%)	ST131 n = 26 (%)	ST1284 n = 18 (%)	ST167 n = 17 (%)	ST405 n = 11 (%)	Other STs[#] n = 112 (%)	All STs n = 229 (%)
Geographic location	Global	Global	Turkey	Global	Global	Global	Global
QRDR mutations							
gyrA S83L	44 (98)	26 (100)	18 (100)	17 (100)	10 (91)	76 (68)	191 (83)
gyrA D87N	44 (98)	25 (96)	18 (100)	17 (100)	10 (91)	61 (54)	175 (77)
parC S80I	44 (98)	25 (96)	18 (100)	17 (100)	11 (100)	62 (55)	177 (78)
parC E84V	0[a]	24 (92)[b]	0[a]	0[a]	0[a]	1 (1)	25 (12)
parE I529L	0[a]	25 (96)[b]	0[a]	0[a]	0[a]	1 (1)	26 (11)
Carbapenemases							
KPC-2	3 (7)[a]	8 (31)[b]	0[a]	0[a]	3 (27)	21 (19)	35 (15)
KPC-3	0[a]	7 (27)[b]	0[a]	0[a]	0	7 (6)	14 (6)
KPC-18	0	1 (4)	0	0	0	0	1 (0.4)
NDM-1	0[a]	4 (15)[b]	0	3 (18)	0	12 (11)	19 (8)
NDM-4	0	0	0	0	1 (9)	0	1 (0.4)
NDM-5	12 (27)[a]	1 (4)[b]	0[b]	11 (65)[c]	3 (27)	13 (12)	40 (17)
NDM-6	0	0	0	0	0	1 (1)	1 (0.4)
NDM-7	1 (2)	0	0	0	0	4 (4)	5 (2)
OXA-48	2 (4)	2 (8)	0	0	4 (36)	32 (29)	40 (17)
OXA-181	21 (47)[a]	1 (4)[b]	18 (100)[c]	2 (12)[b]	0[b]	6 (5)	48 (21)
OXA-232	0	1 (4)	0	0	0	4 (4)	5 (2)
OXA-244	0	0	0	0	0	3 (3)	3 (1)
VIM-1	0	0	0	0	0	2 (2)	2 (1)
VIM-23	2 (4)	0	0	0	0	0	2 (1)
IMP-59	0	0	0	0	0	2 (2)	2 (1)
NDM-1 + VIM-1	0	1 (4)	0	0	0	0	1 (0.4)
NDM-1 + OXA-181	0	0	0	0	0	2 (2)	2 (1)
NDM-5 + OXA-48	0	0	0	1 (6)	0	0	1 (0.4)
NDM-5 + OXA-181	4 (9)	0	0	0	0	1 (1)	5 (2)
NDM-5 + OXA-232	0	0	0	0	0	2 (2)	2 (1)
Other β-lactamases*							
OXAs	21 (47)[a]	11 (42)[a]	17 (94)[b]	7 (41)[a]	3 (27)[a]	23 (21)	82 (36)
CMYs	40 (89)[a]	2 (8)[b]	18 (100)[a]	0[b]	2 (18)[b]	18 (16)	80 (35)
DHA-1	0	0	0	0	0	3 (3)	3 (1)
CTX-Ms	38 (84)[a]	13 (50)[b]	11 (61)	13 (77)	9 (82)	58 (52)	142 (62)

Characteristic	ST410 n = 45 (%)	ST131 n = 26 (%)	ST1284 n = 18 (%)	ST167 n = 17 (%)	ST405 n = 11 (%)	Other STs[#] n = 112 (%)	All STs n = 229 (%)
TEM-1	35 (78)[a]	18 (69)[a,c]	0[b]	8 (47)[c,d]	3 (27)[b,d]	79 (71)	143 (62)
TEMs (ESBLs)	1 (2)	0	0	1 (6)	0	2 (2)	4 (2)
SHV-2	0	0	0	0	0	1 (1)	1 (0.4)
SHV-12	0	2 (8)	0	2 (12)	0	8 (7)	12 (5)
Aminoglycoside modifying enzymes							
aadA1	0[a]	2 (8)	0	1 (6)	3 (27)[b]	23 (21)	29 (13)
aadA2	15 (33)[a]	1 (4)[b]	0[b]	8 (47)[a]	3 (27)	13 (12)	40 (17)
aadA2b	0	0	0	0	0	6 (5)	6 (3)
aadA22	0[a]	0[a]	0[a]	5 (29)[b]	0	0	5 (2)
aadA5	27 (60)[a]	11 (42)[a]	18 (100)[b]	7 (41)[a]	5 (46)[a]	34 (30)	102 (45)
aadA16	0	0	0	0	1 (9)	4 (4)	5 (2)
aac(3')-IIa	2 (4)[a]	2 (8)[a]	18 (100)[b]	1 (6)[a]	2 (18)[a]	18 (16)	43 (19)
acc(3')-IId	31 (69)[a]	3 (12)[b]	0[b]	2 (12)[b]	1 (9)[b]	20 (18)	57 (25)
aac(6')-Ib	0	0	0	0	0	4 (4)	4 (2)
aac(6')-Ib-cr	41 (91)[a]	6 (23)[b]	17 (94)[a,c]	11 (65)[c,d]	5 (46)[b,d]	41 (37)	121 (53)
aac(6')-Ia	0	0	0	0	0	1 (1)	1 (0.4)
aac(6')-IIa	0	0	0	0	0	1 (1)	1 (0.4)
aac(6')-33	0	1 (4)	0	0	0	1 (1)	2 (1)
ant(2')-1a	0	1 (4)	0	0	0	5 (5)	6 (3)
aph(3')-Ia	0	1 (4)	0	0	1 (9)	6 (5)	8 (3)
aph(3')-Ib	15 (33)[a]	5 (19)[a]	17 (94)[b]	4 (24)[a]	1 (9)[a]	16 (14)	58 (25)
aph(3')-VI	0	2 (8)	0	1 (6)	0	7 (6)	10 (4)
aph(4')-1a	0	0	0	3 (18)	0	2 (2)	5 (2)
aph(6')-1d	34 (76)[a]	7 (27)[b]	14 (78)[a]	5 (29)[b]	6 (55)	38 (34)	104 (45)
rmtB	0[a]	0[a]	0[a]	7 (41)[b]	1 (9)	7 (6)	15 (7)
rmtC	0	2 (8)	0	0	0	3 (3)	5 (2)
Other AMR determinants							
fosA	0	0	0	3 (18)	0	4 (4)	7 (3)
qnrA1	0	1 (4)	0	0	0	2 (2)	3 (1)
qnrB1	0	0	0	0	0	4 (4)	4 (2)
qnrB2	0	0	0	0	0	1 (1)	1 (0.4)
qnrB19	0	0	0	0	0	5 (5)	5 (2)
qnrB4	0	0	0	0	0	3 (3)	3 (1)
qnrB6	0	0	0	0	0	3 (3)	3 (1)
qnrB9	0	0	0	0	0	2 (2)	2 (1)
qnrS1	26 (58)[a]	4 (15)[b]	18 (100)[c]	3 (18)[b]	1 (9)[b]	18 (16)	70 (31)
dfrA1	0	1 (4)	0	0	0	5 (5)	6 (3)
dfrA12	16 (36)[a]	1 (4)[b]	0[b]	8 (47)[a]	3 (27)	17 (15)	45 (20)
dfrA14	0	0	0	0	1 (9)	18 (16)	19 (8)
dfrA15	0	0	0	0	0	1 (1)	1 (0.4)
dfrA17	35 (78)[a,c]	11 (42)[b]	18 (100)[a]	8 (47)[b,c]	8 (73)	33 (30)	113 (49)
dfrA27	0a	1 (4)	0	0	3 (27)[b]	6 (5)	10 (4)
dfrA5	0	0	0	0	0	3 (3)	3 (1)
dfrA7	0	1 (4)	0	0	0	0	1 (0.4)
dfrA8	0	0	0	0	0	1 (1)	1 (0.4)
sul1	41 (91)[a]	15 (58)[b]	18 (100)[a]	15 (88)	8 (73)	63 (56)	160 (70)
sul2	34 (76)[a]	8 (31)[b]	18 (100)[c]	8 (47)[a,b]	5 (46)[a,b]	40 (36)	113 (49)
sul3	0	0	0	1 (6)	0	13 (12)	14 (6)
tetA	9 (20)[a,b]	9 (35)[a,c]	0[b]	12 (71)[c]	5 (46)[a,c]	27 (24)	62 (27)
tetB	35 (78)[a,c]	2 (8)[b]	4 (22)[b,c]	1 (6)[b]	5 (46)[c]	35 (32)	82 (36)
tetD	0	1 (4)	0	0	0	2 (2)	3 (1)
Plasmid types							
Col-like	44 (98)[a]	12 (46)[b]	18 (100)[a]	6 (35)[b]	5 (46)[b]	47 (42)	132 (58)
IncFIA	44 (98)[a]	16 (62)[b]	18 (100)[a]	17 (100)[a]	9 (82)	39 (35)	143 (62)
IncFIB	45 (100)[a]	24 (92)[a]	18 (100)[a]	4 (24)[b]	10 (91)[a]	83 (74)	184 (80)
IncFIC	0	0	0	0	0	10 (9)	10 (4)
IncFII	45 (100)[a]	25 (96)[a,c]	7 (39)[b]	14 (82)[c]	10 (91)[a,c]	71 (63)	172 (75)
IncI1	6 (13)[a]	3 (12)[a]	18 (100)[b]	10 (59)[c]	6 (55)[c]	30 (27)	73 (32)
IncH12	0	0	0	0	0	8 (7)	8 (3)
IncA	0	0	0	0	0	2 (2)	2 (1)
IncB/O/K/Z	0	1 (4)	0	0	0	8 (7)	9 (4)
IncC	2 (4)	4 (15)	0	0	0	13 (12)	19 (8)
IncL	2 (4)[a]	0[a]	0[a]	0[a]	4 (36)[b]	20 (18)	26 (11)
IncM	0	0	0	1 (6)	0	4 (4)	5 (2)
IncN	4 (9)	2 (8)	0	0	4 (36)	14 (13)	24 (10)
IncP	0	4 (15)	0	0	0	1 (1)	5 (2)
IncQ	32 (71)[a]	0[b]	0[b]	0[b]	1 (9)[b]	15 (13)	48 (21)
IncR	2 (4)	2 (8)	0	3 (18)	1 (9)	8 (7)	16 (7)

Characteristic	ST410 n = 45 (%)	ST131 n = 26 (%)	ST1284 n = 18 (%)	ST167 n = 17 (%)	ST405 n = 11 (%)	Other STs[#] n = 112 (%)	All STs n = 229 (%)
IncX1	1 (2)	1 (4)	0	0	1 (9)	5 (5)	8 (3)
IncX3	27 (60)[a]	2 (8)[b]	18 (100)[c]	10 (59)[a]	1 (9)[b]	23 (21)	81 (35)
IncX4	2 (4)	2 (8)	0	3 (18)	0	5 (5)	12 (5)
IncX5	0	0	0	0	0	1 (1)	1 (0.4)
IncU	0	0	0	0	0	2 (2)	2 (1)
IncY	9 (20)	0	0	0	0	12 (11)	21 (9)

QRDRs; quinolone resistance-determining regions;

[a-d]Rates followed by different letters indicate significant differences between dominant STs at the 5% level (adjusted for multiple comparisons for each outcome);

[#]Other STs (no) include the following: ST10 (5), ST12 (2), ST34 (1), ST38 (10), ST44 (1), ST46 (5), ST58 (2), ST69 (3), ST73 (1), ST88 (1), ST95 (4), ST127 (1), ST141 (1), ST156 (3), ST191 (1), ST205 (1), ST244 (1), ST345 (1), ST349 (1), ST354 (7), ST357 (2), ST361 (9), ST404 (1), ST448 (4), ST538 (1), ST540 (1), ST607 (1), ST617 (4), ST624 (1), ST635 (1), ST648 (8), ST973 (1), ST1148 (1), ST1193 (2), ST1196 (1), ST1431 (1), ST1434 (1), ST1470 (1), ST1487 (1), ST1722 (1), ST2003 (2), ST2083 (2), ST2172 (1), ST2279 (1), ST3580 (1), ST4553 (1), ST5229 (1), ST6802 (1), ST11260 (6);

*Other β-lactamases (no) include the following: OXA-1 (66), OXA-9 (11), OXA-10 (5), CMY-2 (40), CMY-4 (4), CMY-6 (4), CMY-16 (1), CMY-42 (30), CMY-146 (1), CTX-M-3 (2), CTX-M-8 (1), CTX-M-9 (1), CTX-M-14 (8), CTX-M-15 (103), CTX-M-24 (12), CTX-M-27 (6), CTX-M-55 (8), CTX-M-196 (1), TEM-33 (1), TEM-35 (1), TEM-52 (1), TEM-143 (1).

Appendix Table 2. Virulence profiles associated with dominant Sequence Types among carbapenemase-producing *Escherichia coli*

Characteristic	ST410 n = 45 (%)	ST131 n = 26 (%)	ST1284 n = 18 (%)	ST167 n = 17 (%)	ST405 n = 11 (%)	Other STs[#] n = 112 (%)	All STs n = 229 (%)
Adhesions							
papA	0[a]	21 (81)[b]	0[a]	0[a]	4 (36)[c]	24 (21)	49 (21)
papC	1 (2)[a]	10 (38)[b]	0[a]	0[a]	2 (18)	20 (18)	33 (14)
iha	0[a]	20 (77)[b]	0[a]	0[a]	0[a]	18 (16)	38 (17)
fimH	45 (100)	26 (100)	18 (100)	17 (100)	11 (100)	112 (100)	229 (100)
tsh	0	0	0	0	0	4 (4)	4 (2)
hra	11 (24)[a]	14 (54)[b]	0[c]	15 (88)[d]	9 (82)[b,d]	40 (36)	89 (39)
afaABCD	0[a]	5 (19)[b]	0	0	0	15 (13)	20 (9)
draABCDP	0	0	0	0	0	0	0
Toxins							
hlyA	1 (2)	0	0	0	0	23 (21)	24 (10)
sat	0[a]	21 (81)[b]	0[a]	0[a]	0[a]	17 (15)	38 (17)
vat	0	0	0	0	0	15 (13)	15 (7)
astA	0[a]	2 (8)[a]	18 (100)[b]	0[a]	0[a]	13 (12)	33 (14)
cnf1	0[a]	7 (27)[b]	0[a]	0[a]	0	5 (4)	12 (5)
Siderophores							
iroN	1 (2)	0	0	0	0	27 (24)	28 (12)
fyuA	15 (33)[a]	26 (100)[b]	0[c]	9 (53)[a]	11 (100)[b]	64 (57)	125 (55)
ireA	0	1 (4)	0	0	0	9 (8)	10 (4)
iutA	7 (16)[a]	20 (77)[b]	18 (100)[b]	4 (24)[a]	3 (27)[a]	48 (43)	100 (44)
Capsular antigen							
kpsM II	0[a]	18 (69)[b]	0[a]	0[a]	7 (64)[b]	36 (32)	61 (27)
kpsMT III	0[a]	0[a]	0[a]	0[a]	4 (36)[b]	1 (1)	5 (2)
Miscellaneous							
usp	0[a]	26 (100)[b]	0[a]	0[a]	0[a]	23 (21)	49 (21)
traT	8 (18)[a]	24 (92)[b]	18 (100)[b]	15 (88)[b]	10 (91)[b]	71 (63)	146 (64)
ompT	1 (2)[a]	26 (100)[b]	0[a]	2 (12)[a]	0[a]	48 (43)	77 (34)
iss	3 (7)[a]	25 (96)[b]	18 (100)[b]	17 (100)[b]	0[a]	56 (50)	119 (52)
malX	0[a]	26 (100)[b]	0[a]	0[a]	0[a]	16 (14)	42 (18)
cdtB	0	0	0	0	0	0	0
cvaC	1 (2)	0	0	0	1 (9)	20 (18)	22 (10)
ibeABC	0	0	0	0	0	11 (10)	11 (5)
Virulence scores*	2 (1–7)[a]	11 (8–15)[b]	5 (5–5)[c]	5 (4–6)[c]	5 (3–7)[c]	6 (1–16)	5 (1–16)

papAC, genes of P fimbriae operon; *iha*, iron-regulated adhesin; *fimH*, type-1 fimbriae; *tsh*, temperature sensitive hemagglutinin; *hra*, heat-resistant agglutinin, *afa,* afimbrial adhesin; *dra*, Dr binding adhesins; *hlyA*, α-hemolysin; *sat*, secreted autotransporter toxin; *vat*, vacuolating autotransporter toxin; *astA*, enteroaggregative *E. coli* toxin; *cnf1*, cytotoxic necrotizing factor; *iroN*, salmochelin (siderophore) receptor; *fyuA*, yersiniabactin (siderophore) receptor; *ireA*, iron-regulated element (catecholate siderophore); *iutA*, aerobactin (siderophore) receptor; *kpsM* II, group II capsule variants synthesis; *kpsM* III, group III capsule variants synthesis; *usp*, uropathogenic-specific protein; *traT*, complement inhibition protein; *ompT*, outer membrane protein T; *iss*, increased serum survival; *malX*, pathogenicity island marker; *cdtB,* cytolethal distending toxin B; *cvaC*, factor facilitating colonization; *ibeABC,* invasion of brain endothelium;

[a-d]Rates followed by different letters indicate significant differences between dominant STs at the 5% level (adjusted for multiple comparisons for each outcome);

*The virulence gene score was the number of virulence operons detected (median; range).

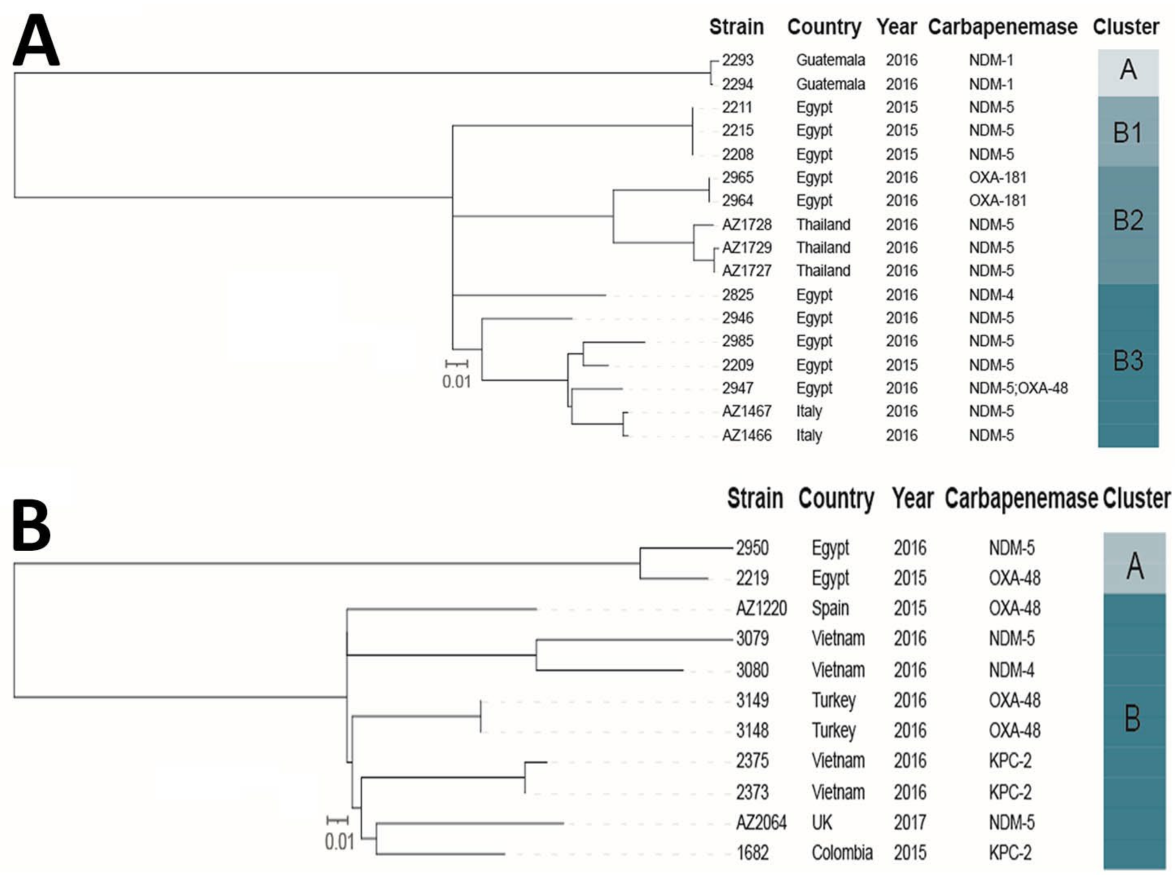
A
Strain
Country
Year
Carbapenemase
Cluster
2293 Guatemala 2016 NDM-1
2294 Guatemala 2016 NDM-1
2211 Egypt 2015 NDM-5
2215 Egypt 2015 NDM-5
2208 Egypt 2015 NDM-5
2965 Egypt 2016 OXA-181
2964 Egypt 2016 OXA-181
AZ1728 Thailand 2016 NDM-5
AZ1729 Thailand 2016 NDM-5
AZ1727 Thailand 2016 NDM-5
2825 Egypt 2016 NDM-4
2946 Egypt 2016 NDM-5
2985 Egypt 2016 NDM-5
2209 Egypt 2015 NDM-5
2947 Egypt 2016 NDM-5;OXA-48
AZ1467 Italy 2016 NDM-5
AZ1466 Italy 2016 NDM-5
A
B1
B2
B3
0.01
B
Strain
Country
Year
Carbapenemase
Cluster
2950 Egypt 2016 NDM-5
2219 Egypt 2015 OXA-48
AZ1220 Spain 2015 OXA-48
3079 Vietnam 2016 NDM-5
3080 Vietnam 2016 NDM-4
3149 Turkey 2016 OXA-48
3148 Turkey 2016 OXA-48
2375 Vietnam 2016 KPC-2
2373 Vietnam 2016 KPC-2
AZ2064 UK 2017 NDM-5
1682 Colombia 2015 KPC-2
A
B
0.01
